# Gleanings

**Published:** 1897-02

**Authors:** 


### GLEANINGS.

One gains some idea of the advance in the craft of horseshoeing and how well those who are directing these movements for a better education of the master-shoers of the future, by noting the subjects suggested for consideration at their meeting by the Committee on Science and Literature of the national body:

1. Corns: how to shoe for them. 2. Quarter and toe-cracks: how to shoe for them. 3. Side-bones, and how to shoe for them. 4. Seedy toe: its cause and how to shoe for it. 5. Over-reaching: its cause and how to prevent. 6. Interfering: the cause and how to prevent (hind foot). 7. Interfering: the cause and how to prevent (front foot). 8. Knuckling: the cause and how to shoe to prevent. 9. Stumbling: the cause and how to shoe to prevent. 10. Hopping behind: the cause and how to shoe to prevent. 11. Cross-firing and how to shoe to prevent. 12. The tip: its use and abuse. 13. The bar shoe: its use and abuse. 14. The side-weight shoe. 15. The toe-weight shoe. 16. The feather-edge shoe. 17. The roller-motion shoe. 18. Calks: their use and abuse. 19. Rubber pads: their uses. 20. The best rubber pad in use. 21. Hot *vs.* cold fitting. 22. What is a pumiced foot, and how to shoe for it. 23. Levelling and preparing the foot. 24. Handling and securing the kicking horse. 25. How the pacer should be shod. 26. How the trotter should be shod. 27. How the runner should be shod. 28. How the roadster should be shod. 29. How the hunter should be shod. 30. How the combination horse should be shod. 31. How the fire-patrol horse should be shod. 32. How the police-patrol horse should be shod. 33. How the horse should be shod for the farm. 34. How horses should be shod for the lumber woods. 35. How horses should be shod for the mines. 36. How horses should be shod for ice-racing. 37. How to preserve the foot in a natural condition. 38. The never-slip shoe: its advantages. 39. Why does the art of farriery need legislation? 40. What are the benefits of legislation? 42. The scientific horseshoer. 43. How to elevate the craft.

Veterinarians James A. Waugh, of Pittsburg, and William J. Waugh, of the U. S. Army, report additional satisfactory results with the actual cautery in fistulous withers.

A case of equine rabies is reported by Messrs. Carter and Waugh, of Pittsburg. The case followed the bite of a rabid dog some time previously.

The addition of flowers of sulphur to the salt fed to cattle is considered by some an effective method of warding off black-leg in cattle. Sufficient is incorporated to give a yellow coloring to the mixture.

The number of sheep in the world is estimated at 550,000,000. Over one-third of this number are said to be merinos. The United States and Canada make up 46,500,000 of this total.

Dr. Nieman, bacteriologist, of Berlin, Germany, claims the discovery of a new cure for tuberculosis, being the serum from nanny-goats' blood mixed with bacilli of the disease.

The *Revue Vétérinaire* reports that a case of generalized tuberculosis occurring in a she-goat was recorded at the Lyons Veterinary College.

Dr. Roux, in a recent letter bearing upon mallein, makes the following deductions: 1. The dose of mallein solution is 2.5 cc. for a horse of medium weight. 2. In considering the reaction indicating glanders it is necessary to take into consideration the rise in temperature, the local œdema, and the general condition of the horse. 3. When a horse indicates complete reaction inject 2.5 cc. at intervals of about one month. The horse may be considered cured when he has shown no reaction at two consecutive injections. 4. All horses treated in this way are not cured, the treatment being effective only upon those which have but slight lesions. 5. The animals under treatment should be isolated.

Thirty-four thousand four hundred and sixty-two horses were shipped from the United States to Europe during the first nine months of 1896.

Chloral hydrate and bromide of potash combined in watery solution give excellent results in controlling epileptiform seizures in dogs that so often arise from intestinal irritation. A generous dose of sweet or castor oil in the interim of attacks is of advantage.

Some interest is being taken in experiments that are being conducted by Prof. Cossar Ewart in the breeding of what may be called hybrid horses. The experiments have been instituted primarily with a view of throwing light on the theory of telegony. Last year the professor procured from the Zoölogical Gardens, Antwerp, one of the three Burchell zebra sires that are in Europe with a view of having him mated with mares of different breeds. Much difficulty was encountered in getting the zebra mated with mares, and only one foal has yet been produced. The dam of this foal is Mulatto, a young mare lent by Lord Arthur Cecil for the experiment. Mulatto is a jet-black pony of the breed known as the Island of Rum ponies. The foal, which was born on the 12th ult., is a very pretty creature, the characteristic stripes being fawn-colored on a background which is nearly black. If Mulatto's next foal by an equine sire shows any of the characteristic markings of the zebra, then the theory of telegony will have been established.

Veterinarian Waugh, of Pittsburg, reports four cases of tympanites where the escaping gas on abdominal paracentesis burned with a flame when touched with a lighted match in three of the four cases. Those where the gas burned recovered, the other one died. The stomach-trocar was used in one case very effectually.

New York City has decreased her death-rate nearly one-half in the past five years. Much of this gain is credited to a better and purer milk-supply and the use of sterilized milk in children of tender years.

